# Bridging the Gap in Sjögren's Disease: A Comprehensive Review of Unmet Needs, Diagnostic Challenges, and Emerging Therapeutic Strategies

**DOI:** 10.7759/cureus.113186

**Published:** 2026-07-22

**Authors:** Thanda Aung

**Affiliations:** 1 Rheumatology, University of California Los Angeles, David Geffen School of Medicine, Los Angeles, USA

**Keywords:** essdai, high risk lymphoma, sicca, sjogren's disease, unmet need

## Abstract

Sjögren's disease (SjD) is a chronic, systemic autoimmune disease characterized by lymphocytic infiltration of exocrine glands and substantial extraglandular involvement. It is widely recognized as one of the most prevalent systemic rheumatic autoimmune diseases; however, patients experience a diagnostic delay averaging roughly five to six years from symptom onset, during which irreversible glandular and systemic damage may accumulate. No disease-modifying therapy is currently approved for SjD, and management remains largely symptomatic, centered on artificial tears, sialogogues, and hydroxychloroquine, none of which reliably arrest systemic immunopathology. This review synthesizes unmet needs across the diagnostic, symptomatic, and therapeutic domains of SjD, with particular attention to the persistent burden of fatigue, cognitive dysfunction, chronic pain, and lymphoproliferative risk. It examines the clinimetric utility of the European Alliance of Associations for Rheumatology (EULAR) Sjögren's Syndrome Disease Activity Index (ESSDAI) and the EULAR Sjögren's Syndrome Patient Reported Index (ESSPRI) as complementary tools that capture systemic organ-based activity and patient-reported symptom burden, respectively. Their growing role as primary endpoints in modern clinical trials is also discussed. Finally, emerging therapeutic strategies are highlighted, including B-cell/BAFF-receptor-targeted ianalumab, the FcRn blocker nipocalimab, and the CD40-CD40L pathway inhibitor dazodalibep, which have demonstrated promising efficacy signals in randomized controlled trials. Improving outcomes in SjD will require earlier recognition, more consistent application of validated outcome measures in routine practice, and rational adoption of emerging immunoselective therapies guided by biomarker-based patient stratification.

## Introduction and background

Sjögren's disease (SjD), historically termed Sjögren's syndrome, is a chronic autoimmune disorder characterized by lymphocytic infiltration of the lacrimal and salivary glands, producing the hallmark sicca complex of keratoconjunctivitis sicca and xerostomia [1,2]. This review focuses on primary SjD, the prototypic form of the disease, unless otherwise specified. SjD is no longer regarded as a localized "sicca syndrome" but rather as a systemic autoimmune disease capable of affecting nearly every organ system, including the lungs (interstitial lung disease), kidneys (tubulointerstitial nephritis), peripheral and central nervous systems, and the hematologic system, where it confers a markedly increased risk of non-Hodgkin lymphoma compared with the general population [2,3]. In 2025, an international Delphi consensus process formally recommended replacing the term "Sjögren's syndrome" with "Sjögren's disease," recognizing it as a distinct systemic autoimmune disease and discouraging the routine use of the historical primary/secondary classification [1].

Despite its systemic burden, SjD remains profoundly underdiagnosed and undertreated. Population-based and cross-sectional cohort data indicate diagnostic delays averaging approximately five to six years from symptom onset, with a reported mean diagnostic latency of 5.98 years (median two years) in a German cohort. Longer delays correlate with worse self-reported general health and greater symptom burden, including gastrointestinal symptoms and vaginal dryness [4]. Earlier population-based data from Taiwan similarly demonstrated a substantial lag between onset of sicca symptoms and formal diagnosis using national insurance claims data [5]. This “diagnostic odyssey” is compounded by the heterogeneity of clinical presentation, frequent overlap with other connective tissue diseases, and the historically narrow framing of SjD as predominantly a glandular or ophthalmologic condition rather than a multisystem disease [4-6].

A second, equally consequential unmet need is therapeutic: despite decades of clinical investigation, no biologic or systemic disease-modifying antirheumatic drug (DMARD) has received regulatory approval for SjD, and management continues to rely on symptomatic measures such as artificial tears, punctal plugs, sialogogues (pilocarpine, cevimeline), and hydroxychloroquine, none of which have been convincingly shown to alter systemic disease trajectory [3,7]. The 2020 European Alliance of Associations for Rheumatology (EULAR) recommendations for the management of Sjögren's syndrome with topical and systemic therapies reflect this gap, providing predominantly low- to moderate-grade evidence for symptomatic interventions and reserving systemic immunosuppression and biologic agents for organ-threatening extraglandular manifestations on a largely off-label basis [7]. The 2025 British Society for Rheumatology guideline on the management of adult and juvenile-onset Sjögren disease likewise underscores the paucity of high-quality, disease-modifying evidence and the heavy reliance on extrapolation from other autoimmune diseases [8].

This review aims to (1) characterize the principal unmet needs in SjD across diagnostic, symptomatic, and therapeutic domains; (2) clarify the clinimetric role and practical utility of the ESSDAI and ESSPRI instruments in clinical trials and routine practice; and (3) summarize the maturing late-stage therapeutic pipeline, providing practicing rheumatologists with a framework for navigating the transition from purely symptomatic management toward targeted, immunoselective biologic therapies.

Relevant English-language publications were identified through PubMed/MEDLINE, Embase, and Google Scholar through June 2026, with emphasis on recent guidelines, clinical trials, systematic reviews, and landmark studies related to SjD.

## Review

Epidemiology and the burden of disease

SjD is widely cited as the second most prevalent systemic autoimmune rheumatic disease after rheumatoid arthritis, although true population prevalence estimates vary considerably depending on the classification criteria and case-ascertainment method applied. Estimates derived from rigorous population-based studies using current classification criteria place the prevalence of primary SjD in the range of approximately 0.01-0.05%, whereas broader physician-diagnosed estimates used by patient advocacy groups are substantially higher, illustrating the persistent ambiguity created by inconsistent diagnostic criteria and underrecognition in primary care [9,10]. A 2024 American College of Rheumatology abstract analysis estimated a 2024 United States prevalence of approximately 10.6 per 10,000 for physician-diagnosed disease compared with only 1.98 per 10,000 for clinically confirmed cases applying current classification criteria, a discordance that highlights both overdiagnosis in some settings and underdiagnosis in others [10]. SjD predominantly affects women, with a female-to-male ratio frequently cited near 9:1, and most commonly presents in the fourth to sixth decades of life, although pediatric-onset disease, while rare, is increasingly recognized and associated with even greater diagnostic delay and a distinct phenotype dominated by fatigue, arthralgia, and glandular swelling rather than classic sicca symptoms [11].

The diagnostic odyssey: causes and consequences of delay

Several converging factors explain the prolonged diagnostic interval characteristic of SjD. First, sicca symptoms are insidious and frequently normalized by patients and primary care providers alike; objective salivary hypofunction may precede clinically apparent sicca symptoms by a substantial margin. Second, the serological and clinical overlap between SjD and other connective tissue diseases, particularly systemic lupus erythematosus and rheumatoid arthritis, often leads clinicians to anchor on an alternative diagnosis. Third, access to confirmatory testing, including minor salivary gland biopsy, ocular staining scores, and unstimulated salivary flow measurement, is unevenly distributed and underutilized outside specialized rheumatology or oral medicine centers [4,5,9].

The clinical consequences of delayed diagnosis are not merely academic. Longer diagnostic latency has been associated with significantly poorer self-reported general health, greater healthcare utilization, and a higher burden of systemic and extraglandular symptoms at the time of eventual diagnosis [4]. In children and adolescents, diagnostic delay of more than three years from symptom onset has similarly been associated with a significantly higher prevalence of established dryness symptoms at diagnosis, suggesting that earlier recognition could meaningfully alter the trajectory of glandular damage even in younger populations [11]. These findings collectively argue for incorporation of validated screening tools and a lower threshold for rheumatology referral in patients presenting with unexplained sicca symptoms, fatigue, or polyarthralgia, particularly in the presence of positive antinuclear antibodies or anti-Ro/La antibodies.

The systemic and symptomatic burden: fatigue, cognitive dysfunction, and pain

Although SjD is classically defined by glandular dryness, patients consistently rank fatigue, cognitive dysfunction ("brain fog"), and chronic widespread pain among their most disabling symptoms, frequently rating these domains as more burdensome than dryness itself [3,8]. These symptoms correlate poorly with conventional measures of glandular dysfunction or systemic organ involvement, and their pathophysiology is incompletely understood, though proposed mechanisms include central and peripheral cytokine-mediated neuroinflammation, small-fiber neuropathy, autonomic nervous system dysfunction, and disrupted hypothalamic-pituitary-adrenal axis signaling [3]. Importantly, fatigue and pain in SjD are not reliably captured by the ESSDAI, which is weighted toward objective systemic organ involvement, underscoring why patient-reported instruments such as the ESSPRI were developed as a complementary, rather than redundant, measure (discussed further below) [12,13].

Management of this symptomatic triad remains almost entirely non-pharmacologic and supportive, comprising graded exercise, cognitive-behavioral strategies, sleep hygiene optimization, and treatment of comorbid conditions, such as fibromyalgia, depression, and obstructive sleep apnea, which frequently coexist with and amplify SjD-related fatigue [3,8]. 

Lymphoproliferative risk and other extraglandular manifestations

Among the systemic complications of SjD, the risk of non-Hodgkin lymphoma, particularly mucosa-associated lymphoid tissue (MALT) lymphoma arising within affected salivary glands, represents one of the most clinically consequential unmet needs, as no validated pharmacologic strategy reliably prevents lymphomagenesis in high-risk patients [2,3]. Recognized risk factors include persistent salivary gland enlargement, palpable purpura, low C4 complement levels, cryoglobulinemia, and monoclonal gammopathy, and several of these features are explicitly incorporated into the ESSDAI biological and hematological domains, reinforcing the importance of structured, longitudinal disease activity assessment rather than symptom-directed visits alone [2,12,13]. Other extraglandular manifestations contributing substantially to morbidity include interstitial lung disease, peripheral and autonomic neuropathy, cutaneous vasculitis, and renal tubulointerstitial disease, each of which may be clinically silent in early stages and is therefore liable to be missed without systematic, instrument-based screening [2,3].

Clinimetric tools: utility of the ESSDAI and ESSPRI

A central theme uniting the diagnostic and therapeutic unmet needs in SjD is the historical absence of validated, standardized outcome measures suitable for both clinical trials and routine practice. This gap has been substantially addressed by the development of two complementary EULAR-endorsed instruments: the EULAR Sjögren's Syndrome Disease Activity Index (ESSDAI) and the EULAR Sjögren's Syndrome Patient Reported Index (ESSPRI).

The ESSDAI, first described in 2010, is a clinician-completed, weighted composite index assessing systemic disease activity across twelve organ-specific domains: constitutional, lymphadenopathy, glandular, articular, cutaneous, pulmonary, renal, muscular, peripheral nervous system, central nervous system, hematologic, and biological (immunological) domains [12]. Each domain is scored according to a graded activity scale (typically none, low, moderate, or high activity), and domain scores are weighted by clinical importance and summed to produce a total score ranging from 0 to 123, with higher scores reflecting greater systemic disease activity. The ESSDAI has been validated as sensitive to change and capable of discriminating between active and inactive systemic disease, and analysis of 921 Spanish patients within the GEAS-SS registry demonstrated its utility in characterizing the pattern and prevalence of systemic involvement across a large, real-world primary SjD cohort [13]. 

The ESSPRI, developed in 2011, is a brief, three-item patient-reported instrument capturing the severity of dryness, fatigue, and pain (joint and muscle pain) over the preceding two weeks, each scored on an 11-point (0-10) numerical rating scale, with the total ESSPRI score calculated as the mean of the three domain scores [14]. Subsequent validation work jointly assessing the ESSDAI and ESSPRI confirmed both instruments' sensitivity to change and established clinically meaningful improvement thresholds, while also demonstrating that the two measures capture largely non-overlapping dimensions of disease: ESSDAI reflects clinician-assessed, objective systemic organ activity, whereas ESSPRI reflects the subjective symptomatic burden that patients themselves identify as most impactful on quality of life [15]. This dissociation has important clinical implications, as patients with low ESSDAI (objectively quiescent systemic disease) frequently report persistently high ESSPRI scores driven by fatigue and pain, a pattern now explicitly recognized in symptom-based cluster analyses that stratify SjD patients into systemic-activity-dominant versus symptom-burden-dominant phenotypes. In routine clinical practice, regular application of both instruments, even in abbreviated form, allows clinicians to distinguish patients who require escalation of systemic immunosuppressive therapy (high ESSDAI) from those whose unmet need is predominantly symptomatic (high ESSPRI with low ESSDAI), informing fundamentally different management strategies and helping to set realistic expectations regarding which emerging therapies are most likely to benefit a given patient phenotype [12-15].

Emerging therapeutic frontiers

The therapeutic landscape for SjD has shifted markedly over the past several years, moving from a near-exclusive reliance on symptomatic and off-label immunosuppressive therapy toward a maturing pipeline of immunoselective biologic agents, several of which have now reported positive phase 2 or phase 3 data.

BAFF/BAFF-Receptor Blockade: Ianalumab

Ianalumab is an afucosylated IgG1 monoclonal antibody with a dual mechanism of action, combining enhanced antibody-dependent cellular cytotoxicity-mediated B-cell depletion with direct blockade of the B-cell activating factor receptor (BAFF-R), thereby targeting both existing autoreactive B cells and the survival signaling that sustains them [16,17]. Recent late-stage clinical studies have demonstrated clinically meaningful improvements in systemic disease activity, supporting further development of ianalumab as a promising therapy for SjD [16,17]. In the paired, global phase 3 NEPTUNUS-1 (N = 275) and NEPTUNUS-2 (N = 504) trials, ianalumab 300 mg administered subcutaneously monthly met its primary endpoint in both studies, demonstrating a statistically significant improvement in ESSDAI change from baseline at week 48 compared with placebo, representing the first phase 3 programs in SjD to demonstrate statistically significant improvement in systemic disease activity. Secondary endpoints, including Patient and Physician Global Assessment scores, also showed numerical improvement, and the safety profile was comparable to placebo [17-19].

FcRn Blockade: Nipocalimab

Nipocalimab is a fully human monoclonal antibody that selectively blocks the neonatal Fc receptor (FcRn), preventing IgG recycling and thereby reducing circulating IgG autoantibodies, including disease-associated antibodies such as anti-Ro52 and anti-Ro60, without broadly suppressing protective immunity. In the phase 2 DAHLIAS trial, a global, double-blind, placebo-controlled study enrolling 163 anti-Ro-seropositive adults with moderate-to-severe SjD, nipocalimab met its primary endpoint with a statistically significant improvement in ClinESSDAI score at week 24 compared with placebo, accompanied by a dose-dependent reduction in total IgG exceeding 77% at the highest studied dose, and numerical improvements in patient-reported dryness, fatigue, and joint pain [20]. These findings support a mechanistic link between autoantibody burden and systemic disease activity and have informed initiation of the phase 3 DAFFODIL program [20].

CD40-CD40L Costimulation Blockade: Dazodalibep

Dazodalibep is a novel non-antibody fusion protein that functions as a CD40 ligand antagonist, blocking the CD40-CD40L costimulatory interaction between T cells and CD40-expressing B cells and antigen-presenting cells, a pathway implicated in ectopic germinal center formation and class-switched autoantibody production within SjD-affected glands. In a phase 2, randomized, double-blind, placebo-controlled crossover trial enrolling two distinct SjD populations, dazodalibep met its primary endpoint in both cohorts: a significant reduction in ESSDAI in patients with moderate-to-severe systemic disease activity (least-squares mean change -6.3 versus -4.1 with placebo; p = 0.0167), and a significant reduction in ESSPRI in patients with high symptom burden but limited systemic organ involvement (-1.8 versus -0.5 with placebo; p = 0.0002) [21]. Dazodalibep also significantly reduced serum CXCL13 and rheumatoid factor levels, biomarkers reflective of germinal center activity, and was generally well tolerated, without the thrombotic signal previously observed with some anti-CD40L antibodies that bind the FcγRIIa-activating Fc domain [21].

Together, these three programs illustrate convergent but mechanistically distinct strategies for interrupting the B-cell-centric immunopathology of SjD (Table 1). Their differing primary endpoints-ESSDAI-driven for ianalumab and nipocalimab, and dual ESSDAI/ESSPRI-driven for dazodalibep-also exemplify the practical trial-design utility of the clinimetric framework discussed above. Other agents under earlier-phase investigation, including the anti-CD40 monoclonal antibody iscalimab and additional FcRn- and BAFF-pathway-targeting agents, further reflect the breadth of immunoselective approaches now being pursued for a disease that, until recently, lacked any late-stage disease-modifying candidate [16,22].

**Table 1:** Selected Emerging Therapeutic Agents in Sjögren's Disease BAFF, B-cell activating factor; CFB, change from baseline; ClinESSDAI, Clinical ESSDAI (biological domain excluded); CXCL13, C-X-C motif chemokine ligand 13; ESSDAI, EULAR Sjögren's Syndrome Disease Activity Index; ESSPRI, EULAR Sjögren's Syndrome Patient Reported Index; FcRn, neonatal Fc receptor; RF, rheumatoid factor This table is adapted from published clinical trial data on ianalumab, nipocalimab, and dazodalibep [17-21].

Agent	Mechanism	Trial/Phase	Primary Endpoint Met	Key Findings
Ianalumab	Afucosylated anti-BAFF-R mAb; B-cell depletion + BAFF-R blockade	Phase 3 (NEPTUNUS-1/2)	Yes - ESSDAI CFB at week 48	Statistically significant ESSDAI reduction in both replicate trials; favorable safety; FDA Breakthrough Therapy designation (2026)
Nipocalimab	Anti-FcRn mAb; reduces IgG/autoantibody recycling	Phase 2 (DAHLIAS)	Yes - ClinESSDAI at week 24	≥77% IgG reduction at 15 mg/kg; numerical improvement in dryness, fatigue, pain; published in The Lancet (2025); phase 3 DAFFODIL ongoing
Dazodalibep	CD40 ligand antagonist (fusion protein); blocks T-B costimulation	Phase 2 crossover (two cohorts)	Yes - ESSDAI (pop 1); ESSPRI (pop 2)	Significant reduction in both ESSDAI and ESSPRI across two distinct phenotypic populations; reduced CXCL13/RF; well tolerated

Limitations and future directions

Several gaps remain even as the therapeutic and clinimetric landscape matures. First, no validated biomarker reliably predicts which patients will respond to a specific mechanism of action; baseline autoantibody titers have shown promise in nipocalimab trials but require prospective validation [20]. Second, the relative contributions of central sensitization, fibromyalgia overlap, and true SjD-specific neuroinflammation to the fatigue-pain-cognitive dysfunction triad remain incompletely disentangled, complicating both trial design and patient counseling [3,8]. Third, real-world implementation of ESSDAI and ESSPRI outside clinical trials remains inconsistent, and abbreviated or digitally administered versions may be needed to facilitate routine adoption in busy rheumatology practices [12-15]. Finally, as multiple late-stage agents approach potential approval, head-to-head comparisons and biomarker-guided sequencing studies will be required to rationally position these therapies relative to one another and to existing off-label immunosuppressive strategies [16].

## Conclusions

SjD remains a significant unmet medical need spanning diagnostic, symptomatic, and therapeutic domains. Persistent diagnostic delay, an underrecognized burden of fatigue, cognitive dysfunction, and pain, and the historical absence of approved disease-modifying therapies have collectively limited the quality of care available to patients. The complementary application of ESSDAI and ESSPRI provides a validated framework for distinguishing objective systemic disease activity from patient-reported symptom burden, informing both clinical trial design and individualized management decisions. The recent positive phase 2 and phase 3 results for ianalumab, nipocalimab, and dazodalibep represent a potential inflection point, offering the prospect of the first approved, mechanistically targeted disease-modifying therapies for SjD. Practicing rheumatologists should incorporate validated disease activity and patient-reported outcome measures into routine longitudinal assessment in preparation for an era of biomarker-guided, immunoselective therapies that may become part of standard SjD management.

## References

[1] Ramos-Casals M, Baer AN, Brito-Zerón MD (2025). 2023 International Rome consensus for the nomenclature of Sjögren disease. Nat Rev Rheumatol.

[2] Brito-Zerón P, Baldini C, Bootsma H (2016). Sjögren syndrome. Nat Rev Dis Primers.

[3] Baldini C, Fulvio G, La Rocca G, Ferro F (2024). Update on the pathophysiology and treatment of primary Sjögren syndrome. Nat Rev Rheumatol.

[4] Meinecke A, Kreis K, Olson P (2024). Impact of time to diagnosis in patients with primary Sjögren's syndrome: a cross-sectional study. Clin Exp Rheumatol.

[5] Huang YT, Lu TH, Chou PL, Weng MY (2021). Diagnostic delay in patients with primary Sjögren's syndrome: a population-based cohort study in Taiwan. Healthcare (Basel).

[6] Vivino FB, Carsons SE, Foulks G (2016). New treatment guidelines for Sjögren's disease. Rheum Dis Clin North Am.

[7] Ramos-Casals M, Brito-Zerón P, Bombardieri S (2020). EULAR recommendations for the management of Sjögren's syndrome with topical and systemic therapies. Ann Rheum Dis.

[8] Price EJ, Benjamin S, Bombardieri M (2025). Executive summary: British Society for Rheumatology guideline on management of adult and juvenile onset Sjögren disease. Rheumatology (Oxford).

[9] Qin B, Wang J, Yang Z, Yang M, Ma N, Huang F, Zhong R (2015). Epidemiology of primary Sjögren's syndrome: a systematic review and meta-analysis. Ann Rheum Dis.

[10] Izmirly PM, Buyon JP, Wan I (2019). The incidence and prevalence of adult primary Sjögren's syndrome in New York County. Arthritis Care Res (Hoboken).

[11] Zeng W, Thatayatikom A, Winn N (2024). The Florida Scoring System for stratifying children with suspected Sjögren's disease: a cross-sectional machine learning study. Lancet Rheumatol.

[12] Seror R, Ravaud P, Bowman SJ (2010). EULAR Sjogren's syndrome disease activity index: development of a consensus systemic disease activity index for primary Sjogren's syndrome. Ann Rheum Dis.

[13] Ramos-Casals M, Brito-Zerón P, Solans R (2014). Systemic involvement in primary Sjogren's syndrome evaluated by the EULAR-SS disease activity index: analysis of 921 Spanish patients (GEAS-SS Registry). Rheumatology (Oxford).

[14] Seror R, Ravaud P, Mariette X (2011). EULAR Sjogren's Syndrome Patient Reported Index (ESSPRI): development of a consensus patient index for primary Sjogren's syndrome. Ann Rheum Dis.

[15] Seror R, Theander E, Brun JG (2015). Validation of EULAR primary Sjögren's syndrome disease activity (ESSDAI) and patient indexes (ESSPRI). Ann Rheum Dis.

[16] Patel V, Salas A, Grader-Beck T (2026). Clinical trials and new therapies in Sjögren's disease. Curr Opin Immunol.

[17] Thomas GB, Xavier M, Stephanie F (2026). Ianalumab demonstrates significant reduction in disease activity in patients with Sjögren’s disease: Efficacy and safety results from two global Phase 3, randomized, placebo-controlled double-blind studies (NEPTUNUS-1 and NEPTUNUS-2). ACR Convergence 2025. Abstract presented at the American College of Rheumatology Annual Meeting; October 24-29, 2025; Chicago, IL, USA..

[18] (2026). Two-arm Study to Assess Efficacy and Safety of Ianalumab (VAY736) in Patients With Active Sjogren's Syndrome (NEPTUNUS-1). https://clinicaltrials.gov/study/NCT05350072.

[19] (2026). Three-arm Study to Assess Efficacy and Safety of Ianalumab (VAY736) in Patients With Active Sjogren's Syndrome (NEPTUNUS-2). https://clinicaltrials.gov/study/NCT05349214.

[20] Noaiseh G, Sivils KL, Campbell K (2025). Efficacy and safety of nipocalimab in patients with moderate-to-severe Sjögren's disease (DAHLIAS): a randomised, phase 2, placebo-controlled, double-blind trial. Lancet.

[21] St Clair EW, Baer AN, Ng WF (2024). CD40 ligand antagonist dazodalibep in Sjögren's disease: a randomized, double-blinded, placebo-controlled, phase 2 trial. Nat Med.

[22] Fisher BA, Szanto A, Ng WF (2020). Assessment of the anti-CD40 antibody iscalimab in patients with primary Sjögren's syndrome: a multicentre, randomised, double-blind, placebo-controlled, proof-of-concept study. Lancet Rheumatol.

